# The socializing role of the physical education teacher at school: what can we learn from the purposes of socialization?

**DOI:** 10.3389/fsoc.2024.1377496

**Published:** 2024-04-10

**Authors:** Bechir Nasri, Ines Souid

**Affiliations:** ^1^Higher Institute of Sport and Physical Education of Kef, University of Jendouba, Béja, Tunisia; ^2^University of Lyon, Lyon 1 L-VIS EA 74 28 SFR CRIS FED 4272, France

**Keywords:** socialization, interaction, social integration, school culture, teaching practice

## 1 Introduction

Our opinion article presents a point of view on the process of socialization for social actors and the socializing role of physical education teachers at school. As a subject taught and particularly as a social vector, physical education, or in other words, team spirit and letting off steam, leads to changes in the individual's perception of themselves and others and thus proves to be a very interesting discipline. The impact of socialization through physical education is significant and can play a major role in preparing the new generations of students for socio-professional integration in a country where change among young people is very important.

Physical education allows students to acquire a range if varied skills, including verbal, physical, and relational abilities, which is not the case in classrooms where apprentices remain seated and silent to take other subjects (Talbot, 2011). The originality of physical education lies above all of the physical activity and multiple, motor, communicative, and emotional expressions. The teacher must adapt to the context of his teaching, as each educational institution has a specificity depending on the school population it welcomes.

We present our opinion about the teaching practice of teachers. First, we present the role of social origin and training in their profound influence of socialization (Nasri et al., 2022). Then, we seek to understand the socialization of teachers and social workers, which takes place through collaboration in schools through education and training and in their orientation and experiences within the organization of the school (Bronstein, 2003). Drawing on recent socialization publications, we evaluate through this opinion article the incorporation of specific case studies and recent qualitative data to enrich our analysis.

## 2 Social interactions shape behavior

A recent analysis focused on the socializing role of physical education teachers at the Lille academy in France. The study underlines that the teacher, within the framework of his socializing missions and his teaching context, defines his teaching content without adopting a precise strategy. In addition, in this art of resourcefulness, he makes the effort to persevere in his being (Darmon, 2010). In this context, another study addresses the importance of deepening scientific and formative knowledge to raise teachers' awareness of the treatment of diversity and intercultural and interpersonal relationships and put into practice the socialization mission (Gayet, 1998).

Over time, values in society crumble, and teachers who are aware of being role models for students must therefore pay attention to their behavior, practices, and actions. Whether consciously or not, they can also pass on to students a taste for school.

At the start of the 2006 school year, an analysis was carried out in ~249 French middle schools that were in the greatest difficulty. Students indulge in behaviors such as absenteeism, incivility, and dropping out. A real socialization challenge then arises for them, to which teachers attempt to respond by strengthening their authority in class and occasionally abandoning their learning requirements. This longitudinal case study analyzes the classroom activity of an experienced teacher and his students during gymnastics cycles. The results show that the teacher installs a silent socialization process by creating synergy between school socialization and the transmission of knowledge about gymnastics (Gal-Petitfaux and Vors, 2019).

On the other hand, teachers can anchor and instill in the heads of their students a disgust for school and teaching, which can further be cultivated by the students to the extent that they receive messages from their teachers negatively. However, during the teaching process, teachers use non-verbal communication, such as movements and postures, to convey their messages and information to students. In this way, children learn—indirectly—in the educational process.

Among the possible approaches to this problem, a recent observation is the analysis of legislative measures in France for disabled children. The school's teaching team insists that the integration of disabled children be presented as a part of the school's project. The teacher, whose effectiveness can depend on several factors including training, relationships with students, and commitment to continuous improvement, relied on his training and experience in the school system, as well as the knowledge of psychology acquired during his university studies (Éric and Cornelia, 2013).

A central role should be given to the socializing role of teachers and to learn to influence. It is necessary to inculcate in their cognitions the skills of creative thinking, which could well contribute to improving educational and social conduct in a changing society. In addition, the difficulties of certain adolescents suffering from emotional and intellectual problems are reflected negatively in their school life. A significant disparity is evident in the cultural attitudes and societal expectations across different countries, particularly between Arab and Western nations, in terms of fostering individual creativity. In Arab countries, there appears to be less encouragement or support for individuals to fully harness their creative potential. Conversely, in Western countries, such abilities are generally nurtured and promoted. This divergence in cultural norms and societal values contributes to a pronounced disparity in the cultivation and appreciation of creativity across these regions.

In this sense, Mennesson explains that children would have a tendency to be influenced in their choices of practices by the parents of the same sex as them. Extending this idea, this finding would imply that students could have a tendency to get involved depending on their gender and physical education activities (Mennesson, 2011).

## 3 The socializing role through experiences

First of all, there are organizations and associations that offer activities allowing young people to improve their cultural, political, and social skills and life in general with the aim of building human consciousness. Just like the agents of socialization discussed previously, these environments are governed by precise norms, roles, and statuses. Cloutier and Drapeau (2008) confirm the distinction between two types of socialization through leisure: structured activities and unstructured activities.

Another research explored the link existing between socialization and creativity during adolescence. The study examined 149 French adolescents and 173 Syrian adolescents. The objective was to assess whether the relationships between creativity and socialization are analogous in the two cultural contexts and not to compare the creative performances of the two groups of adolescents. In addition, the objective was to evaluate separately the development of creative thinking in the two populations. Overall, the correlations between creativity and socialization turned out to be rather weak except for the perception of academic success and the practice of extracurricular activities. The analysis showed that the evaluation of creativity depends on cultural representations and acquired experience (Pacteau and Lubart, 2005).

The metaverse is still a relatively new concept, but it is rapidly evolving in how people socialize and connect with each other in the future. These platforms are becoming more user-friendly and accessible, making it easier for individuals to participate in shared virtual experiences and form communities based on common interests. These platforms mirror real-world events like concerts and sporting events, providing new opportunities for socialization and community building (Prolitus Technologies, 2023).

## 4 The generational transmission of social behavior

Professional socialization, by which the members of a particular community of workers progressively internalize the values, norms, symbols, models, roles, and knowledge that are privileged, or imposed by their reference group, or by that of belonging, exerts a very great influence on their opinions and attitudes, while imprinting on their behavior a specific direction, form, and content. The fact that these people have several things in common also facilitates communication and allows them to commune with certain feelings to share aspirations, tastes, needs, and activities, in short, to share together enough ideas and particular traits to recognize oneself.

According to some studies, the physical education (PE) class can enchant a student's day, but it can also become a weekly nightmare for others. Indeed, the worst memories of PE students are often linked to a feeling of embarrassment experienced in physical practice, a lack of interest in the activities offered, or even the difficulty of portraying one's body (Marlière, 2013). In fact, these challenges have a direct link with the identity of the physical education subject, which is characterized by a motor aspect, unlike other cognitive subjects. On the other hand, each society or country has its own culture and social and intellectual structures, and as a result, the subjects taught are often ranked according to the importance of the content of each subject taught. Therefore, developed countries do not have differences in the level of classification compared with other non-developed countries in terms of education.

A recent qualitative analysis aims to compare two countries, one belonging to the Western world and the other to the Arab world. The analysis results underlined the differences between Western societies, in particular French society and Syrian society, which are quite broad in terms of socialization. These divergent ideas on socialization and personal accomplishment do not prevent a community of thought regarding the specific role of society in the development of the child. It is useful to identify the positive aspects of this period in both societies so as to bring about favorable developments without major difficulties. We would like to know what it is because only a few studies are interested in this subject. National education systems are the product of different histories, philosophies, and legal and political contexts, and all relevant variables must be taken into account for comparison (Fischer, 2010).

On the other hand, the construction of knowledge and a particular physical education identity allows for a better understanding of the substance of the test in question. The peer group in a professional world contributes to learning new educational trends in line with the real demands of our society and to our world of work. This problem of cohesion in the way of directing students' practice or on the content provided can have an influence on the educational imbalance encountered in class. These teachers are then obliged to carry out certain reorganizations to establish and make students accept their own mode of operation.

In 2015, UNESCO developed physical education guidelines to improve policies and practices for quality physical education across all age groups globally. One of the tasks of those responsible for education and teaching programs is to create PE programs with creative content, rich in skills, consistent with social reality and more adapted to the needs of students and social changes to improve their behavior within society (UNESCO, 2015).

Finally, to learn to influence through the teaching of physical education and to strengthen the socializing role of the teacher in the class, it is necessary to emphasize certain skills such as the integration of speaking (with movements and gestures). In addition, it is necessary to closely link theory and practice.

As a conclusion, it also seems relevant to recall the words of Bourdieu (1998), who considers that the work of socialization, carried out from childhood, continued and reinforced by the sporting environment, imposes limits that all concern the body. It thus creates tastes, choices, talents, and behaviors different for each sex (Boutin, 2009). Obviously, this study has its limits that it is up to us to correct our exploration of the socializing role of physical education teachers in schools, in addition to the limited number of published research that focuses on the notion of socialization to enrich knowledge made on the basis of socialization. The strength of this study remains the attachment to innovative work closely associated with all stages of human life.

## Author contributions

BN: Writing – original draft. IS: Writing – review & editing.
